# 

**Published:** 1895-08

**Authors:** 


### CONTENTS.

ORIGINAL COMMUNICATIONS—

REPORT OF SURGICAL CASES. By W. S. Armstrong, M.D., Atlanta, Ga. .... 321

OBSERVATIONS ON THE VISUAL FIELDS AND EARLY FUNDUS CHANGES IN RETINITIS PIGMENTOSA. By Alex. W. Stirling, M.D., Atlanta, Ga. .... 328

VITAL STATISTICS, ETC., AND THE HYGIENE OF THE CONVICTS, HOSPITAL, AND PRISON CAMPS OF THE PENITENTIARY OF GEORGIA, FROM OCTOBER 1, 1890, TO OCTOBER 1, 1894. By W. O'Daniel, M.D., Bullard, Ga. .... 336

THE MANAGEMENT OF HEMORRHAGE AFTER TONSIL OPERATIONS. By A. W. Calhoun, M.D., LL.D., Atlanta, Ga. .... 340

SOCIETY REPORTS—

CINCINNATI OBSTETRICAL SOCIETY .... 343

CORRESPONDENCE—

NEW YORK LETTER .... 350

EDITORIAL—

ARE WE DRIFTING IN OUR ETHICS? .... 356

AMICK *vs.* REEVES .... 357

THE LATE PROFESSOR HUXLEY .... 358

SELECTIONS AND ABSTRACTS—

SKIN DISEASES AND SYPHILIS .... 360

GENERAL MEDICINE AND THERAPEUTICS .... 363

PRACTICAL CHEMISTRY AND PHARMACY .... 379

MEDICAL ITEMS .... 381

BOOK REVIEWS .... 382

ADVERTISERS' INDEX TO ADVERTISEMENTS .... xii

MEDICAL ITEMS—CONTINUED .... x, xi

CLASSIFIED INDEX TO ADVERTISEMENTS .... xxxv

